# Using lessons learnt from key stakeholders to increase support for scaling the Reach Up Early Childhood Parenting program

**DOI:** 10.3389/fpubh.2023.1151826

**Published:** 2023-08-08

**Authors:** Jacqueline Coore-Hall, Joanne Smith, Melissa Kelly, Helen Baker-Henningham, Susan Chang, Susan Walker

**Affiliations:** ^1^Epidemiology Research Unit, Caribbean Institute for Health Research, The University of the West Indies, Kingston, Jamaica; ^2^World Bank, Washington, DC, United States; ^3^School of Human and Behavioural Sciences, Bangor University, Bangor, United Kingdom

**Keywords:** Reach Up program, scale-up, global community, program resources and materials, key informants, implementation research

## Abstract

**Introduction:**

Sustainable implementation of early childhood programs requires resources, materials and methods that are adaptable, scalable and feasible for delivery through multiple sectors. Additional or modified program resources may be required to meet emerging needs, as programs go to scale. An active and effective monitoring, evaluation and learning (MEL) process may enable programs to be responsive to demands. The Reach Up: Early Childhood Parenting program, is designed primarily for disadvantaged children under 4 years of age in low- and middle-income countries (LMICs) to promote their development through playful caregiver interactions. The curriculum, training manuals and other materials and resources support implementers in the adaptation of the intervention, implementation, workforce training, monitoring and evaluation. This paper reports on how data collected from key informants drove modifications to program processes, materials and resources.

**Methods:**

We conducted in-depth interviews with 14 key informants (including program managers, lead trainers, academics, consultants and workforce personnel) on their experiences with Reach Up across 15 LMICs where the program has been implemented. We also reviewed written records generated from (i) structured small group discussions at a Knowledge Exchange meeting of 31 Reach Up partners and (ii) notes from working groups formed at the meeting and tasked to continue working post-meeting to find solutions to support ongoing implementation. The transcripts from the in-depth interviews and the meeting records were analysed using thematic analysis with a mixture of pre-defined categories and data-driven sub-themes.

**Results:**

The main findings indicated that there was a need to: (i) develop advocacy and communication resources and materials to aid prospective implementers and other stakeholders, to make decisions for implementation, (ii) revise and/or add to the content and format of the curriculum and add content in the training and other supporting manuals and (iii) enhance the training process.

**Conclusion:**

The feedback from the key global partners informed the development of new knowledge materials, resources and processes and modifications to existing program materials and resources. These will help to support advocacy, ongoing implementations, and the process of transitioning the Reach Up early childhood intervention to scale.

## Introduction

The *Lancet* early childhood development series in 2016, the launch of the WHO, UNICEF and World Bank *Nurturing Care Framework* in 2018, the special issues of *Annals of the New York Academy* in 2018 and the *Archives of Diseases in Childhood* series in 2019, highlighted the significance of addressing early childhood development (ECD), especially for children in low- and middle-income countries (LMICs). Increasing numbers of LMIC have national ECD polices (1) with increased demand for scalable and sustainable ECD interventions.

To achieve lasting impact on early childhood development and caregiver skills and practices, interventions require a comprehensive package of resources and materials that are adaptable, scalable, promote sustainability, and feasible for implementation through multiple sectors. An active and effective monitoring, evaluation and learning (MEL) system can be important in understanding effective scaling. As Krapels et al., noted, this is needed to (i) facilitate strengthening of programs and inform the development of more effective designs, implementation and scale up and (ii) share information and knowledge gained from MEL processes with others working in the ECD and other behaviour change sectors (2).

The Measurement for Change (M4C) approach provides a framework that can be used to measure progress and respond to developing needs as necessary, to ensure continued effective adaptation and implementation and facilitate scaling of ECD interventions. The five interconnected objectives that currently inform the use of data collected for M4C are that it should be (i) dynamic (flexible to change and use iterative learning cycles), (ii) inclusive (engaging all stakeholders – children, families, community members, policy makers), (iii) informative (making decisions based on the data gathered from various sources), (iv) interactive (continually interacting with systems, processes and stakeholders and implementing changes based on these interactions) and (v) people-centred (be able to respond to the diverse needs of all stakeholders) (2) The framework, with focus on objective (ii) was used as the guiding approach for this study.

Studies on scale up of ECD programs have focused on examining of geographical coverage and reach, implementation characteristics such as dosage, frequency and mode of delivery, monitoring (supervision, fidelity), evaluation of intervention outcomes, financing and the workforce (3). In addition to monitoring implementation characteristics such as coverage, mode of delivery and impact, other elements of an intervention, such as the program material and resources and capacity to implement, may also affect its success. Research on this aspect of scaling ECD programs through evaluating and improving the processes, resources and materials to support achievement of scaling at other levels, is less common.

In this paper, we document lessons learnt from engagement with the Reach Up: Early Childhood Parenting program (commonly referred to as “Reach Up”) implementation partners in multiple countries. We also discuss how this informed enhancements to the Reach Up package to support the needs of program managers and delivery staff as they expand to reach more families.

First, we present a brief overview of the Reach Up program. We then discuss Phase I, the evolution from the Jamaica Home Visit intervention (JHV) to Reach Up. Phase II (the formation of the global Reach Up community) and Phase III (using learnings from the global partners to make systematic changes to further scale the Reach Up package) are discussed in the Results section.

## The Reach Up Early Childhood Parenting program

The Reach Up: Early Childhood Parenting program is based on the Jamaica Home Visiting (JHV) model developed in the 1970s (4–6). It was designed primarily for disadvantaged children under 4 years of age in low- and middle-income countries. In addition to helping parents promote their children’s development, the program aims to build the mother’s self-esteem and enjoyment of parenting (6). The format of delivery of the intervention is through a series of weekly or fortnightly home visits or small group sessions, using a structured curriculum. The activities are tailored to the child’s ability and each play session includes the introduction of concepts (using homemade toys, songs and games) and language activities. The intervention is designed to be suitable for delivery by non-professionals, with a minimum of complete primary level education, such as community health workers and community members (e.g., *Madre Guias* – “mother guides” in Guatemala and Colombia). The initial Reach Up package comprised weekly and fortnightly curricula for ages 6–36 months, an Adaptation and Planning Manual for program implementers, a Toy manual, a Training manual with films to be used during training workshops and a Supervisor Manual. The intervention has been adapted and used in 17 countries: Bangladesh, Bolivia, Brazil, China, Colombia, Guatemala, India, Jamaica, Kenya, Liberia, Madagascar, Peru, Turkey, Zimbabwe and Jordan, Lebanon and Syria (for Syrian refugee families). At scale, in Peru, the national home visiting program, *Cuna Más*, was built on Reach Up, through the adapted Colombian model (7). The intervention has also been expanded to 11 counties in the western and central regions of China (8). The intervention has been adapted and implemented in diverse contexts and settings, such as in poor, rural and urban communities and humanitarian, conflict and displacement settings such as the implementations led by the International Rescue Committee (IRC) in the Middle East.

Adaptations done for context prior to implementation include the inclusion of local games and songs, adaptation of pictures and toys to reflect local people and activities, and addition of content to the curriculum (e.g., health, nutrition and hygiene messages) and training manual (e.g., stress management). Adaptation of implementation processes to fit country infrastructure, personnel and resources include mode of delivery (e.g., weekly to fortnightly visits), personnel (categories of workers used – healthcare workers, community leaders, pre-school teachers), engagement strategies (recruitment of participants, retention of workforce) and training.

Evidence shows that Reach Up can be feasibly delivered through health services or social services reaching families with young children in several countries (9–14). The intervention has been adapted for delivery through small group sessions in India (15), Bangladesh (14, 16) and Colombia (12). The impact of the intervention has been measured through randomized control trials which have shown benefits to child development and parenting outcomes (4, 9, 14–21). Substantial long-term benefits to cognition, education, mental health and income up to age 30 years have also been demonstrated (22, 23).

Evaluations of implementation processes have been conducted in Jamaica (24), Bangladesh (14, 25), Colombia (26) Brazil and Zimbabwe (27) and the Middle East (28). These have included evaluations of the adaptation and implementation processes, focussing on the acceptability, appropriateness, feasibility and effectiveness from the perspectives of caregivers (mothers) and implementation staff.

### Scaling up the Reach Up intervention package

Scaling of the processes, resources and materials that make up the Reach Up intervention has occurred across three phases:

- Phase I, the evolution from the Jamaica Home Visit intervention (JHV) to Reach Up, a comprehensive package of curricula, manuals and resource materials in 2014.
- Phase II, implementation and evaluation in several countries leading to a knowledge exchange meeting and the formation of the global community in 2019
- Phase III, interconnected with Phase II, using learnings from global partners to make systematic changes to further scale the Reach Up package in 2021.

### Phase I: from Jamaica home visit to Reach Up (1973–2014)

Up to 2014 the JHV program was adapted for use in Bangladesh, Colombia (the first attempt to scale the intervention by linking to the conditional cash transfer program *Familias en Acción*) and Peru (adapted and implemented at scale through the *Cuna Más* program) (5). Randomized control trials conducted in Bangladesh (5–7, 29, 30) and Colombia (10), in addition to those in Jamaica (4, 9, 17) provided robust evidence that the program had benefits to children’s development.

### Development of the Reach Up package

In a context of growing demand for scalable ECD programs and an increase in interest in the implementation of the JHV, the developers identified challenges with the expansion related to limited technical capacity of organizations as well as capacity of the Jamaica intervention team to provide support. One of the solutions identified was to develop a comprehensive training package that could be used by implementation partners.

With the support of a grant in 2014 from Grand Challenges Canada, a web-based package was developed. The curriculum layout was reorganized for easier use with drawings of toys and suggested text to introduce and explain activities included. A few new picture books, toys and activities were added to the curriculum to replace some toys and create more variety. The toy manual was revised with colourful drawings depicting step by step instructions. All pictures (used in books and puzzles) were redrawn with the use of vibrant colours to make them more attractive to young children. Three additional manuals to support implementation and training were developed (Adaptation and Planning manual, Training manual and Supervisor training manual). To facilitate the training, 23 short films (approximately 2–3 min each), were produced to show the methods used during visits and demonstrated specific materials and activities. Three 15-min films produced show the key steps in a home visit. All films are available in Bengali, English, French and Spanish.

A meta-analysis of impact evaluations of the JHV and Reach Up in several low- and middle-income countries, showed that the intervention improved child development across diverse settings (31).

## Methods

We focussed on the *inclusive* aspiration of the Measurement for Change (M4C) framework as the intent of this paper is to highlight how learnings from Reach Up partners, who are critical stakeholders, contributed to the scaling of the Reach Up program resources.

The data we present in this paper were collected in Phase II of the Reach Up scale-up process. We used two approaches (i) interviews with key informants on their experiences with Reach Up in the different countries where the program has been implemented and (ii) review of written records generated from meetings with members of the global Reach Up community.

In November 2019, with funding from the LEGO Foundation, we convened a 3-day Knowledge Exchange Meeting to bring together 31 partners to discuss the findings from the intervention, including reflections on the challenges, successes and lessons learned from the implementation of Reach Up. The attendees at the Knowledge Exchange Meeting were program implementers familiar with the processes involved in the decision-making, adaptation and implementation of country-specific interventions, the core team of Reach Up developers, and representatives from the LEGO Foundation, The World Bank and the Inter-American Development Bank who had been involved in supporting the implementation of Reach Up. We also asked country program leads to recommend team members who would be able to contribute to the discussions on country-specific adaptations and implementations.

Before the meeting, three researchers (JC-H, JS, and MK) obtained information from a purposive, non-random sample of 14 key informants over the period August to October 2019. Informants were chosen as they were experienced with the intervention and/or had first-hand knowledge of the processes involved in the decision-making, adaptation, and implementation of country-specific interventions. Reach Up developers identified persons from the existing network of partners who were experienced Reach Up trainers, country program leads who had conducted planning and adaptation of the intervention, or researchers who undertook evaluations of the intervention. We also asked program leads to recommend team members involved in frontline delivery, so that we could capture their “on the ground” experiences. The sample was deliberately restricted as the intention was to interview one key person involved in the planning and implementation of the respective programs, and to invite one or two additional key people to the Knowledge Exchange Meeting. One informant provided information for two separate country programs and two informants provided information for one country, but on different modes of delivery (groups and home visits). All persons who were approached agreed to be interviewed.

Thirteen interviews were conducted in English and one in Spanish, each lasting approximately 1 h. Due to geographical distance, the interviews were held *via* Zoom®. A structured guide consisting of 28 questions was used during the process. The guide was divided into three main sections: Program Design (Adaptations and Program Materials), Implementation (Lessons Learned and Promoting Play) and Overall Reflections. Table 1 presents the questions related to the program design which is the focus of this paper. The participants were not provided with a copy of the interview guide prior or during the actual interview.

**Table 1:** Interview questions specifically on program design.

**Adaptation** In the survey you shared that you adapted the (indicate which – curriculum, training materials, supervision materials, play materials, etc. for families). Can you please share with me how you determined that these adaptations were necessary? Can you please tell me how the adaptations were made, including who was involved in the process (consultants, government officials, trainers, front line workers)? Did you have an opportunity to test/pilot the adaptations of the materials before full implementation? (If yes, continue). What aspects were you able to test/pilot before the start of the intervention?
**Programme materials** What do you think about the Reach Up Curriculum? (e.g., objectives, layout). Do you think anything else needs to be included? Any sections that need to be improved/expanded? What do you think about the Reach Up Toy Making manual? (e.g., instructions, illustrations, measurements) Do you think it needs to be improved/expanded? Can you describe any significant challenges you may have had in getting the toys, blocks, books and puzzles made? What do you think about the Reach Up Adaptation manual? (e.g., instructions for adaptation, advice on information to be collected, etc.) Do you think anything else needs to be included? Any sections that need to be improved/expanded? What do you think about the Reach Up Training process and the Training Manual? (e.g., organisation of sessions, content, layout). Do you think anything else needs to be included? Any sections that need to be improved/expanded? What do you think about the Reach Up Supervisor Guidelines? (e.g., content, descriptions)? Do you think anything else needs to be included? Any sections that need to be improved/ expanded?

The interviews were recorded, and the interviewers also kept written notes throughout the interview. Transcriptions of the recordings were compared with the written notes, compiled, and saved as Microsoft Word documents. The 15 transcripts from the 14 interviews (one informant was interviewed for two separate countries) were numbered R1 through R15. Approval to carry out interviews was received from the Ethics Committee of the University of the West Indies in August 2019 (approval number ECP 3, 19/20). Written consent forms were emailed to the participants for their signature.

We also reviewed written records generated through two major sources (1) the Knowledge Exchange Meeting held in November 2019 where discussions and recommendations were documented and (2) the minutes of meetings from working groups formed at the meeting and tasked to continue working post-meeting to find solutions to support ongoing implementation. Working groups were formed to focus on three key areas – Adaptation, Capacity Building and Monitoring and Evaluation.

### Data analysis

#### Interviews with key informants

The data from the interviews with the key informants were analysed using a qualitative content analysis framework. Specifically, we used thematic analysis, following Braun and Clarke’s (32) recommended five phases for identifying, analysing and reporting patterns within data – (i) familiarization with data, (ii) generating initial codes, (iii) searching for and generating themes, (iv) reviewing themes and (v) defining and naming themes. We applied a mixture of deductive coding (pre-defined categories) and inductive coding (data-driven sub-themes). We first identified nine pre-defined themes based on the main section headings and corresponding questions on the structured interview guide – Adaptation, Integration, Manuals, Curricula, Training Process, Implementation, Workforce, Monitoring and Evaluation and Promoting Play. In this paper, we present the analysis from four of the pre-defined themes related to the Reach Up processes, material and resources – Adaptation, Training process, Manuals, and Curricula.

Two researchers (JC-H and JS), working independently, manually coded nine and six transcripts, respectively. The researchers were currently working with the Reach Up program and therefore understood the context of the review. At this stage, each researcher coded their respective transcripts using the initial codes they identified.

Following the individual coding of the transcripts, the researchers reviewed, and met to discuss each other’s extracted supporting quotes. Any discrepancies were resolved through discussions and consensus and consultation with an arbitrator (SW). Based on the initial coding, we identified 39 sub-themes to which data could be coded, and formulated definitions for each (Table 2). The coded texts were then combined under each category and sub-theme and saved as the final output of the coding process.

**Table 2:** Pre-defined categories and data driven sub-themes identified.

Pre-defined categories	Data driven sub-themes
Adaptation	Culture Stakeholder engagement Delivery mode Piloting of materials
Integration	Types of integrated services Barriers to integration Benefits to integration
Manuals	Layout of the manuals Content of the material
Curricula	Format/structure of the curriculum Content of the curriculum Development of new content and/or curricula
Training process	Quality of lead trainers (train-the-trainers) Duration (length of time) Mode of delivery
Implementation	Stakeholder engagement Relationships with families Communication Working relationship Cultural norms Impact on families Material costs Material procurement Geography and climate Scaling
Workforce	Compensation Supervision Turnover Workload Gender Profile Benefits (of the program) to workforce
Monitoring & evaluation (Supervision)	Structure of supervision Supervision of workforce Importance of supervision and mentorship Training of supervisors
Promoting play	Promoting play through training workshops and material/resources Promoting play during visits Importance/impact of play

#### Review of meeting records

One researcher (JC-H) reviewed the compiled notes from the Knowledge Exchange Meeting and minutes of three of the working group meetings (Adaptation, Capacity Building and Monitoring and Evaluation) to identify discussions surrounding the resources and materials and extract relevant statements, decisions and future actions.

Similar to the coding process for the interviews, the nine pre-defined themes were used in the process. The notes from the Knowledge Exchange Meeting, and the Adaptation, Capacity Building and Monitoring and Evaluation working groups were read and sentences and/or paragraphs were highlighted and labelled with the most appropriate theme according to the codebook.

## Results

Three male and eleven female key informants were interviewed from NGOs, and academic and funding institutions, from various regions across the world (Table 3).

**Table 3:** Summary of key informants.

Participant	Sub-region	Institution/affiliations	Position/involvement in the program
R1	South Asia	Research Institute	Researcher; trainer
R2	South Asia	Research and NGO	Researcher
R3	South America	Academic Institute	Researcher; trainer
R4	South America	Government	Consultant
R5	South America	Government	Consultant; trainer
R6	South America	Government	Consultant; trainer
R7	South America	Academic Institute and Government	Researcher; trainer
R8	Southern Africa	NGO	Program lead
R9	Central America	NGO	Supervisor
R10	East Asia	Research Institute	Program lead
R11	Middle East	NGO	Home visitor
R12	Middle East	NGO	Supervisor; trainer
R13	Southern Europe	Academic and Funding Institution	Researcher; trainer
R14	Caribbean	Government	Program lead
R15	Southern Africa	Academic Institute and Government	Researcher

Findings for Phases II and III of the Reach Up material and resources scale up are presented in this section. For Phase II, we present (i) the background to the formation of the global community and (ii) the systems used to share lessons and make resources accessible to implementation partners. For Phase III, we (i) highlight the learnings from the interviews with the key Reach Up partners and the review of the records of the Knowledge Exchange and working groups’ meetings, and (ii) describe the adaptations made to some of the Reach Up materials and the addition of new knowledge goods and resources.

### Phase II: formation of global Reach Up community

The formation of the global Reach Up community began with the participants who attended the Knowledge Exchange Meeting in November 2019. The group included Reach Up developers, implementation program leads, government representatives, lead trainers and academic researchers from Bangladesh, Bolivia, Brazil, China, Colombia, Guatemala, India, Jamaica, Jordan, Madagascar, Peru, Turkey and Zimbabwe. The 3 days of activities included presentations on experiences with home visiting (four countries) and group modalities (three countries), sharing of results from a pre-meeting survey and a summary of the indepth interviews, in-session working groups (more detailed discussion focussed on adaptation and preparing to implement, workforce capacity, implementation, and learning), gallery walks (comprising notes from working group discussions mounted on flip charts for participants to review and add comments), and other networking activities.

### Knowledge sharing

Since the formation of the global community, we have established systems for sharing learnings with, and making resources available and accessible to, Reach Up partners. Networking and communication among the partners have been strengthened through:

- The knowledge goods produced and the new and amended materials were shared with Reach Up partners at a webinar in November 2021.
- A Bi-annual Newsletter to share information about implementation in the different countries, provide updates on workshops and meetings and links to reports, journal articles, evaluations, information from funding agencies, etc.
- A group was formed on the instant messaging platform, WhatsApp^®^, which included country representatives, implementers, funders and Reach Up team members in Jamaica. The platform has become a place for active exchange of ideas and where members can seek possible solutions for implementation challenges.
- The file hosting service Dropbox^®^ is used to store and organize program documents and resources as they become available. These are made available under the Creative Commons License Attribution-NonCommercial-ShareAlike 4.0 International (CC BY-NC-SA 4.0), to registered Reach Up partners and organizations and are implementing or planning to implement the program. Registered partners and organizations sign a Memorandum of Understanding on the use and citation of the materials, training by experienced trainers, and sharing of new materials.

### Phase III: learnings from key Reach Up partners

The results from the coding of the four categories related to the Reach Up material and resources – Adaptation, Training process, Manuals and Curricula – are presented in this section. Table 4 summarises the key findings from the interviews with the key informants and the discussions at the Knowledge Exchange Meeting and Working Groups, while Table 5 presents some of the perspectives and recommendations from the key informants.

**Table 4:** Key findings from interviews and meeting discussions.

Source	What was learnt
Key informant interviews	Involving decision makers at the government level is a critical component for the effective implementation and eventual scaling of the intervention. And as part of this engagement process, the key informants emphasized that advocacy and communication resources and material must be available. There was a need to make revisions and/or modifications to the curriculum (e.g., develop additional sections with activities) and messages for other age groups (e.g., 0–5 months and 37–48 months). There was also demand for additional content in the supervisor and training material (e.g., additional guidelines for trainers). The training process could be improved with adjustments to the duration and mode of delivery. For example, decrease in the number of training days and the use of technology to deliver virtual training sessions.
Records from the Knowledge Exchange Meeting and working groups	Key priorities/deliverables necessary: Develop key arguments for the intervention based on theoretical framework Preparation of advocacy and communications material that can facilitate global conversations with funders, governments, researchers etc. Develop guidelines and minimum standards for the adaptation of Reach Up. Provide guidelines on integrating Reach Up within existing services such as health and education. Provide guidelines for engaging government and community stakeholders Adjust/strengthen the training and supervisor manuals in areas such as mentoring and coaching, conducting meetings and building positive relationships. Develop model terms of reference document for each staff category Identify tools to assess the skills, characteristics and competencies of the workforce Proposed list of monetary and non-monetary incentives for the workforce Explore solutions for the use of technology – in training, monitoring and supervision

**Table 5:** Examples of some of the perspectives and recommendations for adaptation, curricula, manuals, and training process.

Perspectives/recommendations	Examples of comments
*Stakeholder engagement is key*	*Put together a persuasion strategy to have them [government] buy-in at the end of the day, into the intervention. And perhaps a presentation that would showing benefits, perhaps, then we would have convinced them to implement it at scale*… (R3) *It’s been a challenge to get the “X” city government to conceptually understand the objectives of the methodology…. it would be helpful if some background on the methodology was described clearly*. (R4) *There needs to be prep work on how to present the program to get buy-in. This includes videos, materials. Focus on the outcomes and then show the curriculum. The government needs to understand the essence of Reach Up and buy in on the methodology*. (R5). [Adaptation manual] *should include a community mobilization guide to understand the steps to entering into a partnership with government and community members.* (R9) *Engaging with the government and community stakeholders from the beginning is the key to buy-in and sustainability. This includes … a communication mobilization guide to understand the steps to entering into a partnership with government and community members.* (R9)
*Changes/additions to the curricula*	*Need longer introduction, defining objectives,* etc. *scientific support, information on development.* (R3) *It would be very helpful to have more information about the rationale behind the curriculum and the sequence of activities to show the developmental skills.* (R6) *I think it will be amazing if we can work with families with children at one month. Need curriculum for* [*sic*] *one month.* (R12)
*Additional content in supporting manuals*	[Training manual] *It would be great to see sessions on problem-solving – what to do in real life situations where children are not cooperating or engaged in activities.* (R4) [Training manual] *There should be a module on facilitation skills, including how to be dynamic and to motivate the mothers/caregivers.* (R9) *There should be a section of the training* [manual] *for supervisors/training of trainers to prepare to cascade the training.* (R9) *The training manual should include an overall understanding of ECD, including an overview of child development milestones.* (R10) [Supervisor Guidelines] *Need to include some assessment forms. For example, use some aspects of the early development assessment tool and include skills that the home visitors need to have, like some teaching, communication and feedback skills.* (R11) [Supervisor Guidelines] *The layout of the manual that the supervisor has to use need adaptation to facilitate ease of use in the field.* (R11) [Integration of activities] *into the daily routine, I think that will be very helpful. Given that some families have older children, some activities that can easily incorporate other children can be specified more directly to mothers*. (R13) S*upervisor guidelines need tweaking for the context…look at all the variables that the supervisor must observe and the tool should be user friendly and capture the most essential factors.* (R14)
*Enhancements to the training process*	*In order to improve the training process, the training should be reduced from 10 days since that amount of time is not feasible within a big, integrated program…Before or in between training sessions in person, virtual videos…thus reducing the days of training.* (R5) *It is not viable to have a 10 day training, especially in the case of an integrated program. It is important to think about how to reduce the number of* [training] *days, making some sessions virtual beforehand or in between sessions; Think about technology, how to use online sessions.* (R6) *I think the training can be done in seven to eight days and not ten.* (R11)

### Implementation challenges and suggested solutions

A few key informants identified challenges with integrating the program within an existing system. Cost and increased staff workload were mentioned, but the bureaucratic systems at State-run institutions was the main challenge experienced with integration. Suggestions made by the key informants to engage policy makers and minimize bureaucratic challenges are mentioned in Table 6.

**Table 6:** Examples of some challenges and solutions during adaptation and implementation.

Sub-theme	Examples of comments	
	Challenges encountered	Solutions implemented
Stakeholder engagement	*Getting access to the local health department was difficult.* (R2) *Challenges are balancing with other content needs and bureaucracy.* (R5) *The challenge was that* [XX] *was implementing many programs at the same time for the same families without coordination.* (R4) *Authorities did not want the program because it was not bringing materials/infrastructure.* (R9) *During that time in* [XX] *the ministries, the directors of the ministries were changing a lot, there was not much stability.* (R13)	*Having a local person to support adaptation and liaise with the government to manage voices was helpful.* (R6) *Held workshop with government technical staff to present the curriculum.* (R5) *Community fairs helped them to see the importance of play in children’s growth and development.* (R9) *We used a community mobilization strategy with the Ministry, community leadership for continuous engagement. We saw that the supervisors had too much to do in coaching the home visitors, so we hired supervisors just to engage with government and community stakeholders and to keep them abreast of the project activities.* (R9)
Material Procurement	*Sometimes difficult to find empty water bottles*. (R12) *Getting cardboard given the ban on plastics has made it difficult.* (R14) *Parents thought toys were outdated.* (R10)	*Have recycling containers to collect empty water bottles and provide these for the volunteers to start work with.* (R12) *We have had to use lamination and foam boards.* (R14) *Some toys were replaced with store-bought toys.* (R10)
Supervision	*At the beginning, they had challenges in applying the methodology.* (R10)	*This was resolved through weekly meetings with supervisors, which focused on discussing challenges and building strategies to resolve those challenges.* (R10)
Working relationship	*One major success was the relationship between supervisors and home visitors.* (R4)	*The focus on modelling, reflection, and problem solving helped them to feel supported. They texted their supervisors regularly to problem solve and the supervisors did the same with the coordinator.* (R4)
Cultural norms	*Because of culture they are uncomfortable for anyone to allow male visitors to conduct activities. Most said we need females.* (R12)	*If we have males we prefer to have a female accompany him.* (R12)
Relationship with families	*Even the availability of the mothers, the timing when the mothers were available.* (R15) *A main challenge was getting the mothers to participate because of existing cultural norms-husbands/fathers did not want home visitors to enter the home or the mothers to travel to attend group sessions.* (R3)	*Sometimes the community nutrition worker had to work either in the evenings or very early mornings.* (R15) *Fathers also became very active in toymaking.* (R3)
Workload	*Supervisors complained of heavy workloads since they had to observe every home visitor at least once in a month.* (R10)	*To reduce workload, the program developed an app for their monitoring tools. This helped a lot.* (R10)
Turnover	*High staff turnover because home visitors are volunteers and do not have any benefits.* (R12) *There was some attrition of home visitors which was a significant challenge.* (R13)	*Pay transportation and meals during work.* (R12) *Train more staff than is needed in case you need replacement staff.* (R13)

Adaptation challenges included accessing/finding materials to make the toys, acceptability/suitability of toys and in some instances, costs associated with procuring the required material. The main workforce challenge was staff turnover experienced curing program delivery. Inadequate compensation and heavy workload were the two main factors contributing to staff turnover. Unfortunately, not many solutions were offered, as this issue was often out of the control of the key informants.

Some key informants also noted that the workforce was affected by distance/location of families and the weather. In two countries, challenges related to the safety of the home visitors were of concern:

*It’s a very geographically dispersed setting with the hugest accessibility problem and some security problems. (R15)*

*Difficult working conditions as we had violence and safety concerns in the neighborhoods. (R7)*

Other challenges and suggested solutions by the key informants include supervision, working relationships, cultural norms and relationships with families (Table 6).

### Adaptations/additions and changes to scale the Reach Up package

After the in-depth interviews, further discussions at the Knowledge Exchange Meeting and feedback from the working groups, the following changes to the Reach Up package and resources to support scaling, were made. These were done with input from members of the global community. Tables 7, 8 provide a fuller description of the new knowledge goods produced and the new and amended resources, respectively. In summary these comprise,

**Table 7:** New knowledge goods developed to aid in scaling the Reach Up package.

Advocacy and communication products/resources	
Theoretical background	Focus on the rationale for the Jamaica Home Visit/Reach up design and methods (theoretical background and content of the curriculum).
Theory of change	Provides an overview of the intervention delivery with the inputs needed (including human resources and materials) and the connections between each segment of the intervention. As the program is adapted in each context, the suitable staff needed to deliver the intervention need to be trained and the families identified for intervention. The long-term outcomes include benefits to families and staff as well as capacity building for the organizations who implement the program. The ultimate impact includes benefits to families and children with the developmental potential of these children realized.
Power Point presentation and slide bank	A structured presentation that can be used to support global conversations with main target audience (governments, funders, NGOs) who may be interested in implementing Reach Up. It includes an overview of the program with key principles, evidence, materials. It can be adapted depending on audience with sections more focused towards policymakers and others on implementation. There are additional slides organized by topic (e.g., adapting for context, Reach Up evidence) that can be used as templates for slides in other presentations
Marketing video	A narrated 5-min film on Reach Up highlighting the genesis of the intervention, footage from the training videos, footage of home visits in various countries, evidence from studies conducted, still images from the inception of the JHV on which Reach Up is modeled, information on the package/resources, contact information, etc.
Reach Up brochure and policy sheet (revised versions)	These documents provide a brief overview of Reach Up and summarize the key features of the intervention and how they are related to child development and highlights the benefits of implementing the intervention
Adaptation and planning resources	
Making decisions on integration	Core principles and guidelines for making decisions on integrating the intervention with an existing program such as health and nutrition services, if feasible, as well as guidelines on staffing, supervision, funding, scalability, etc.
Engaging stakeholders	Guidelines on approaches (meetings, sensitization sessions, etc.) to use when seeking support from stakeholders (government and community members) and a checklist of areas/topics to focus on during engagement sessions
What makes Reach Up, Reach Up	Highlights the key components of Reach Up and the essential elements of the intervention which should be part of implementation of Reach Up.
Capacity building resources	
Incentives for workforce	A list of proposed incentives – monetary (e.g., comparable wages/salary, stipend for travel to training/conduct visits/communication), and non-monetary (e.g., exposure to other ECD programs and provision of resources and tools – toys, smart phones/tablets, flyers/bulletins, etc.)
Workforce terms of references	Recommended profile for home visitors and facilitators. Includes education, experience and skills, roles and responsibilities, reporting relationships and demands of the job.
Assessment of skills and competencies	Provides an overview of the expected competences to be achieved from the training, with additional reference to the Early Childhood Workforce Initiative Home Visiting Needs Assessment Tool.

**Table 8:** New and amended program resources and materials.

Resource/manual	Adaptation/additions made to resource/manual
Toy Manual	Additions/revisions to the Toy Manual included: Reorganized by age group and type of toy Improved instructions – include numbered lists of steps to make toys and measurements of materials needed to make the toys Deletion of some toys from the list that were made from materials that are often difficult to source.
Training Manual	The main additions/revisions to the Training Manual were: The “Importance of Play,” referencing LEGO’s playful parenting framework, was added to the introductory section of the manual, to support the rationale for the program. Training session 1 “Introduction to the Programme” also included information and activities on the importance of play and the characteristics of play. Improved sections/statements on working with extended family members, practice activities that include other family members and on how to treat other family members Revised and/or added questions to be asked after viewing training videos, e.g., new questions “What developmental domains does this activity help?” and “What concept words were used?” Added lists of materials by names, descriptions, drawings and age as they appear in the curriculum. Added a new set of practice activities (using blocks and cardboard farm animals) to support the extension of the curriculum for children 36–48 months. A new flip chart with instructions on how to build a positive relationship with the mother/caregiver was included (e.g., sit at the same level, ask their opinions, praise and explain what you want to teach the child and how to do it). Included as Appendices, for ease of reference: Workshop evaluation forms List of role plays by session List of videos by session List of supplemental practice activities
Supervisor Training Manual	The main additions/revisions to the Supervisor Training Manual were: Additional guidelines for the trainer: How to use the manual How to conduct demonstrations and practice activities How to prepare for the training workshops Additional information for training Session 2: Supervisory techniques and methods: Supportive supervision, Building positive relationships Coaching and giving feedback Conducting meetings and field visits Additional information for training Session 3: Responsibilities of a good supervisor Handling community relations Organizing facilitators’ meetings A list of the flip charts required for the training was included as an Appendix for ease of reference.
Supervisor Handbook	A Supervisor Handbook was developed for supervisors who are not train-the-trainers, but who may need guidelines and resources. It covers the following topics/areas: Supervisory techniques Supportive supervision Building positive relationships Coaching and giving feedback Conducting meetings and field visits Individual and Group meetings Field visits/observations Responsibilities of a good supervisor The handbook also includes a list of resources needed for the program and local services that vulnerable families may need and an Observation checklist with clear definitions for each factor
Curriculum 0–5 months 37–48 months	The curriculum was extended to include objectives, activities and resources to be used for visits with children ages 0–5 months and 37–48 months.

- A communication package, developed for advocacy and communication with stakeholders with a modifiable presentation, short film, brochure and policy brief
- Production of new documents and resources including guidelines on integration into existing government systems and workforce related documents with suggested terms of reference, competencies and incentives
- A document on the theoretical background and content of the curriculum was developed and the curriculum extended with content for 0–5 months and 37–48 months (weekly and fortnightly).
- Expansion/revision of training materials including adding a section to enhance the promotion of playful interactions referencing the LEGO Foundation’s characteristics of play and the importance of play for early childhood development (33) and improving sections on working with extended family members.
- The manual for training of supervisors was revised and expanded to include more content on supervisory techniques and methods, with interactive scenarios for mentoring and feedback.
- A Supervisor Handbook was developed to provide guidelines and resources. It covers topics/areas such as supervisory techniques and methods, responsibilities and conducting meetings and field visits.
- Virtual delivery of the train the trainer workshop was hastened by the restrictions on travel and social distancing due to COVID-19. In-person training workshops were suspended, and we pivoted to online delivery in 2021, delivering training content using a mix of synchronous sessions on the Zoom^®^ platform and asynchronous sessions mounted on the Moodle^®^ Learning Management System. We partnered with The University of the West Indies Centre for Excellence in Teaching and Learning to develop the Moodle platform, prepared PowerPoint presentations from the contents of the Reach Up Training Manual and 13 short videos of activities (role plays and materials demonstrations) were produced for use in the virtual training workshop. The synchronous sessions were recorded and uploaded to the Moodle platform daily. The first workshop using virtual delivery was held in June/July 2021 over 10 days, and the second in November/December 2021 reduced to 9 days.

## Discussion

In this paper, we report on how learnings from key stakeholders contributed to the enhancement of Reach Up materials and resources to support effective adaptation and implementation and facilitate scaling the program across new and within existing countries/regions. Information was collected from Reach Up global community stakeholders (consultants, funders, researchers, home visitors and supervisors) who had first-hand knowledge of the processes involved in the decision-making, adaptation, and/or implementation of the intervention.

The Nurturing Care Framework Handbook recommends the formation of communities of practice to facilitate innovation and scale up of ECD interventions (34). The global Reach Up community (officially formed in 2019) is one such network of implementers, program managers and researchers who have led on the initiation, adaptation, implementation, monitoring and evaluation of the intervention in various settings and countries. Similar to others (14, 27, 35, 36) we found that an important part of the adaptation process is the incorporation of feedback from implementers and delivery agents to inform modifications to existing, program material and resources and the development of new resources that can facilitate scale-up.

The expansion of the Reach Up program has been driven by networking, partnerships and collaborations over many years. Some of our partners are champions and leaders of Reach Up through their advocacy and interaction with decision makers (including government policy makers), contributing to adaptations in new countries and/or to extending coverage in countries where the program has already been implemented. Networking at the global community meeting in November 2019 also led to new collaborations and projects between implementers. For example, the International Rescue Committee (IRC) connected with the team from the International Centre for Diarrhoeal Disease Research (ICDDR,b) in Bangladesh to adapt the intervention for Rohingya refugees, and with a team from Bogazici University in Turkey for the Syrian refugee populations in that country.

The feedback from the key stakeholders informed the development of new resources that can be used to present and discuss the features of the intervention with policy makers and other potential users. Content developed include the theoretical background of the curriculum and the theory of change to facilitate communication of the development and mode of action of Reach Up. The PowerPoint presentations and the short film (with footage of training sessions and home visits in various countries) provide target audiences with a better “feel” for the intervention. Overall, the new advocacy products are available to help with communicating and engaging with prospective implementers, including policy makers, community members and funders.

Reach Up is designed to strengthen the capacity of parents and other caregivers to promote the development of their children through interactions with implementation staff who play a key role in the success of the program. Therefore, it is essential that the package provides the content and resources to guide, support and build capacity among the workforce. This was addressed through modifications to the curriculum, training resources and new materials related to workforce competencies and motivation. The Reach Up curriculum was designed to be delivered by facilitators with limited educational qualifications and no special knowledge of children’s development. The inclusion of information from the LEGO Foundation’s Playful Parenting Framework (33) provides facilitators with some theoretical knowledge applicable to the intervention, as well as understanding of benefits of playful parenting in promoting child development.

Key informants feedback also led to improvements in guidance for supervisors/mentors who support frontline delivery staff to maintain quality. The Supervisor Training manual was enhanced with additional content on building positive, respectful relationships, and providing positive feedback for home visitors and a new Supervisor Handbook developed.

Like other ECD programs, challenges remain around retention of staff, especially in contexts where Reach Up is integrated into existing services and is delivered by an existing cadre of workers (24, 35, 37, 37). Competing responsibilities and/or additional duties and lack of motivation (often linked to inadequate compensation and benefits), have been cited as the main contributing factors. While quality relationships, as described above can help to motivate staff, we have now included a list of proposed monetary and non-monetary incentives intended to help program managers develop strategies appropriate for their context.

With restrictions on travel due to the COVID-19 pandemic beginning in early 2020, face-to-face training was suspended and the suggestions from members of the global community for more adaptable train the trainers’ workshops using technology was fast-tracked. Two virtual workshops were held during 2021 and feedback from participants is being used to continue to integrate virtual delivery into training options. Opportunities to shorten the face-to-face training time, using a blended approach with a combination of virtual and face-to-face sessions, and offering the training in phases, are now possible.

We will monitor the use, of the new knowledge goods, through continued engagement and knowledge sharing with stakeholders. Use of a more structured approach to inform scaling of the intervention, was discussed at the knowledge meeting in 2019. For example, in addition to the implementation of the current systems for sharing learnings and making resources accessible (e.g., WhatsApp®, Dropbox®, etc.) the global partners considered development of a Reach Up learning consortium. This would provide a systematic process for what information to collect, how this will be collected, co-ordinated, analysed and reported. An initial step towards developing a learning consortium will focus on within country scaling.

Reach Up is a “living program” and further resources and extensions may be developed informed by continued learnings from implementation partners and other stakeholders. For example, the use of tablet-based applications to support home visitors to select appropriate activities for a child and collect data for program monitoring is being tested in China, Brazil and Jordan and may be important in supporting quality delivery at scale. One extension to date, is the Parent Manual (38) developed in response to the COVID-19 pandemic and demand from partners for ways to continue to provide parenting services. The Parent Manual can be used directly by parents and also provides content from the Reach Up curriculum that can be delivered through text and video messaging, radio and telephone calls. It also includes content on materials in the home that can be used for all activities in this new manual. This addition is important as providing play materials can be a challenge as programs scale.

Experiences with the integration into government services such as the primary care program in Brazil, the nutrition program in Madagascar, Peru’s *Cuna Más* program, Colombia’s FAMI program and ChinaREACH in China, have had mixed results that provide lessons for scaling and suggest large-scale replications of the Reach Up program are possible. The revision and extension of the Reach Up program resources and materials reported here, should help to address some scaling challenges and many of the knowledge goods produced may also be useful for other ECD programs.

The study has a few limitations. There was ongoing networking and interactions prior, during and after projects are implemented, between co-authors and country partners. Therefore, the data may be influenced by social desirability bias. We selected individuals from the existing network of Reach Up partners who were country program leads and trainers. Thus the views of other groups, for example policy makers, front line workers and families in the program, were not included.

This paper illustrates the importance of including the perspectives of key stakeholders in intervention evaluation and using the information in program enhancement so that materials and resources can support scaling. The findings from this study add another dimension to the ongoing debate about “what will be scaled up and how” (39). This case study of Reach Up demonstrates the value of a community of program partners and in-country stakeholders who can exchange experiences and evidence to inform transitioning to large-scale programs.

## Data availability statement

The datasets presented in this article are not readily available because in order to maintain the confidentiality of the key informants, the interview transcripts will not be made available. Requests to access the datasets should be directed to jacqueline.coore@uwimona.edu.jm.

## Ethics statement

The studies involving human participants were reviewed and approved by Ethics Committee, The University of the West Indies, Mona, Kingston, Jamaica. The participants provided their written informed consent to participate in this study.

## Author contributions

JC-H, JS, HB-H, and SW contributed to the conceptual design of the paper. JC-H, JS, and MK were responsible for data collection. JC-H and JS conducted data analysis. SC led the revision of the program materials. JC-H, JS, MK, HB-H, SC, and SW contributed to the interpretation of the findings. JC-H drafted the paper with assistance from all co-authors. All authors contributed to the article and approved the submitted version.

## Funding

The LEGO Foundation supported the Knowledge Exchange Meeting held in November 2019, the conduct of this study and publication. The Foundation was not involved in data collection, analysis, or interpretation.

## Conflict of interest

The authors declare that the research was conducted in the absence of any commercial or financial relationships that could be construed as a potential conflict of interest.

## Publisher’s note

All claims expressed in this article are solely those of the authors and do not necessarily represent those of their affiliated organizations, or those of the publisher, the editors and the reviewers. Any product that may be evaluated in this article, or claim that may be made by its manufacturer, is not guaranteed or endorsed by the publisher.
